# Attitudes towards risk-stratified breast cancer screening: a population-based survey among 5,001 Danish women

**DOI:** 10.1186/s12885-024-12083-2

**Published:** 2024-03-19

**Authors:** Louise Hougaard Loft, Line Hjøllund Pedersen, Janne Bigaard, Stig Egil Bojesen

**Affiliations:** 1 Prevention and Information Dept, Danish Cancer Society, Strandboulevarden 49, DK-2100 Copenhagen, Denmark; 2 Science to Society Dept, Danish Cancer Institute, Danish Cancer Society, Copenhagen, Denmark; 3 Department of Clinical Biochemistry, Herlev and Gentofte Hospitals, Copenhagen University Hospital, Herlev, Denmark; 4 Faculty of Health and Medical Sciences, University of Copenhagen, Copenhagen, Denmark

## Abstract

**Background:**

The individual woman’s risk of being diagnosed with breast cancer can now be estimated more precisely, and screening can be stratified accordingly. The risk assessment requires that women are willing to provide a blood test, additional personal information, to know their risk, and alter screening intervals. This study aimed to investigate Danish women’s attitudes towards risk-stratified breast cancer screening.

**Methods:**

An online, cross-sectional survey was conducted among Danish women aged 52–67 years. We used logistic regression analyses to assess how personal characteristics were associated with the women’s attitudes.

**Results:**

5,001 women completed the survey (response rate 44%) of which 74% approved of risk estimation to potentially alter their screening intervals. However, only 42% would accept an extended screening interval if found to have low breast cancer risk, while 89% would accept a reduced interval if at high risk. The main determinants of these attitudes were age, education, screening participation, history of breast cancer, perceived breast cancer risk and to some extent breast cancer worry.

**Conclusion:**

This study indicates that women are positive towards risk-stratified breast cancer screening. However, reservations and knowledge among subgroups of women must be carefully considered and addressed before wider implementation of risk-stratified breast cancer screening in a national program.

**Supplementary Information:**

The online version contains supplementary material available at 10.1186/s12885-024-12083-2.

## Background

Breast cancer is the most common cancer among women worldwide, accounting for 15% of all cancer-related deaths among women [1]. Although treatment and prognosis have improved considerably in recent decades, the relative survival of breast cancer among Danish women remains lower than that of women in other comparable countries, such as Sweden and Norway [2]. Denmark introduced a national breast cancer screening program in 2008. Women aged 50 to 69 are offered mammography biennially, and 83% of the invited women participate in breast cancer screening [3]. Denmark is a welfare state with free public health care, and all women can participate in screening and be treated free of charge.

Recent research has made it possible to estimate the individual woman’s risk of getting breast cancer using a personal risk score, after which she can be offered an appropriate screening program [4]. Such a risk-stratified breast cancer screening program has the potential to accelerate detection of breast cancer in those women with high risk, reduce false positive and overdiagnosis in women with low risk and overall improve cost-effectiveness of a national screening program [5]. Due to this potential, there is growing interest in the emerging field of risk-stratified breast cancer screening. While no country has fully implemented a national risk-stratified breast cancer screening program, different versions of risk measurements and stratifications are currently being tested in several countries in Europe (My PeBS and BC-Predict) [6, 7], the United States (the WISDOM study) [8] and Canada (PERSPECTIVE I&I) [9]. The randomized trial PRSONAL (Population-based Randomized Study Of a Novel breast cancer risk ALgorithm and stratified screening) will test a risk-stratified breast cancer screening program in the Capital Region of Denmark [10]. The study will recruit 1,000 women, of whom approximately half will receive an individual risk-stratified screening program divided into four risk levels, from low risk to high risk, (using the CE marked CanRisk model [11]), while the other half will continue in the existing national screening program.

Risk-stratified screening is more complex than a one-size-fits-all program. Besides the introduction of new technologies and procedures, risk-stratified screening requires that the women are willing to (1) be made aware of their own risk of developing breast cancer, (2) accept a potential change in the frequency of breast cancer screenings according to their individual risk, (3) provide a blood sample for a genetic analysis, and (4) provide other lifestyle and health information, including personal and family history of breast cancer. In the present study, we explore women’s attitudes towards fulfilling these four requirements.

In many countries, women’s have expressed a high level of interest in risk-stratified breast cancer screening [12–17]. Nevertheless, women also express a degree of hesitancy about extending the interval of their breast cancer screenings if found to be at low risk [14–17]. Previous studies have shown women’s attitudes towards risk-stratified breast cancer screening depends on a range of factors, such as age, education level and history of breast cancer [12, 13, 15, 16]. However, attitudes toward risk- stratified breast cancer screening have not previously been investigated in Denmark.

The objective of this study was to investigate Danish women’s attitude towards risk-stratified breast cancer screening. Based on qualitative research [18], we have hypothesized that women’s attitudes depend on their age, education level, personal or family history of breast cancer, participation in breast cancer screening, perceived risk of breast cancer, and breast cancer worry. Further, we wanted to identify the groups least likely to accept the concept of risk-stratified screening and the possible critique in order to further identify potential barriers and possible facilitators for participation.

## Methods

### Study design and participants

We conducted an online, cross-sectional survey among 5,001 Danish women. Included in the study are women aged 52 to 67 years. The age range was chosen to reflect the current screening target group. Excluded are women who might not have had their first screening mammography e.g., due to postponed screening invitations in recent years (50- and 51-year-old women) and women for whom a risk estimation would not alter their screening program (68- and 69-year-old women). The survey was developed based on knowledge about women’s attitudes toward risk-stratified breast cancer screening obtained from qualitative focus groups and individual interviews [18]. Further, through a literature search, we identified similar survey studies from other countries in order to qualify the survey [12, 13, 15–17, 19]. A pilot test of the survey was conducted among five women in the target group. Convenience sampling was used to identify these five women. This test led to minor adjustments in the wording of the survey. The questions were adapted to a computer assisted web interview and administered by Norstat Denmark [20], a market research firm that uses a web panel from the general population. The web panel received an e-mail with a link to the survey, thus using a non-random sampling. The e-mails were sent out from November 17 to December 8, 2022.

The survey contained 17 groups of questions and took approximately seven minutes to complete. A translation of the survey is presented in Additional file 1. We applied survey quotas on age and region in order to obtain a sufficient number of observations to represent the general Danish female population.

### Measures

Questions from the survey related to women’s willingness to participate in the randomized trial, PRSONAL, are excluded from the present analyses. See Additional file 1 for more details on the questions behind each item described below.

We identified five outcome variables describing attitudes toward risk-stratified breast cancer screening:

1. *Today, the breast cancer screening program is the same for all women aged 50 to 70 years. In the future, it will be possible to estimate the individual woman’s risk of getting breast cancer. What are your thoughts about your personal risk being used to offer you a mammography more often or less often than today?* (That’s a good idea / That’s a bad idea / Don’t know).
2. *What are your thoughts on estimating your risk of developing breast cancer?* (I would like to know my risk / I don’t want to know my risk / Don’t know)
3. *Do you feel comfortable having a blood sample taken to be tested for several breast cancer hereditary genes used to estimate your risk?* (Yes / No / Don’t know)
4. *Imagine that you have had your personal risk of developing breast cancer estimated and are told that you have a high risk. Therefore, you are offered a mammography every year. Which of the following statements best applies to you?* (It would be fine with a mammography every year / I would still prefer a mammography every two years).
5. *Imagine that you have had your personal risk of developing breast cancer estimated and are told that you have a low risk. Therefore, you only need a mammography every four years. Which of the following statements best applies to you?* (It would be fine to get a mammography every fourth year / I would prefer a mammography every two years).

Questions 2 and 3 were dichotomized for the logistic regression analyses (2: I don’t want to know my risk/don’t know vs. I would like to know my risk, 3: No/don’t know vs. Yes). Thus, we compared women who were negative or hesitant with women who were positive.

Determinants in the analyses were:

- *Age*: included in the analyses as a continuous variable.
- *Education level*: included in the analyses as a categorical variable divided into lower (less than 10 years, primary school), medium (10–12 years, upper secondary and vocational) and high education (greater than 12 years, higher education).
- *Screening participation*.
- *Family or personal history of breast cancer*: both included in the analyses as binary variables with the categories “Yes” (oneself or a family member has/have had breast cancer) and “No” or “No/Don’t know”. “Do not wish to answer” was not included in the analyses.
- *Perceived risk of breast cancer*.
- *Breast cancer worry*: For analyses we used three categories: “Almost always/Often”, “Sometimes” and “Rarely/Never/Don’t know”.

### Statistical analysis

We used descriptive statistics to present characteristics of the study population and their attitude towards risk-stratified breast cancer screening. We used multivariable logistic regression analyses to establish the association between three selected variables demonstrating attitude towards risk-stratified breast cancer screening and the independent variables: Age, education, participation in breast cancer screening, history of breast cancer, perceived breast cancer risk and breast cancer worry. All three regression models explored the odds of being negative or hesitant rather than being positive, and each model was adjusted for all seven independent variables. We performed sensitivity analyses to explore potential misclassification bias of combining women who are negative and hesitant by excluding women who are hesitant from the models. We also performed sensitivity analyses, excluding those women who have or have had breast cancer. The results were summarized in odds ratios (ORs) and corresponding 95% confidence intervals (CIs). Statistical analyses were performed using R Statistical software version 4.2.1 [21]. We considered two-sided *P* values of 0.05 or less to indicate statistical significance.

## Results

### Population characteristics

Of the invited 11,391 women, 5,001 (44%) women responded to the survey. The women’s mean age was 59.5 years. Table 1 presents the characteristics of the study population.

**Table 1:** Characteristics of the study population

Characteristics	Total (*n* = 5,001)	%
Age		
52–59	2,578	51.5
60–67	2,423	48.5
Education level		
Lower (< 10 years)	348	7.0^a^
Medium (10–12 years)	2,292	45.8^a^
High (> 12 years)	2,337	46.7^a^
Do you have or have you had breast cancer?		
Yes, I have/have had breast cancer	238	4.8^a^
No	4,752	95.0^a^
Does anyone in your immediate family have or have they had breast cancer?		
Yes, one or more members of my immediate family have/have had breast cancer	1,482	29.6^a^
No / Don’t know	3,507	70.1^a^
Are you participating in the breast cancer screening program, where you are invited every two years?		
Yes	4,609	92.2
No	392	7.8
How do you assess your own risk of getting breast cancer?		
Low	1,717	34.3
Neither high nor low	1,943	38.9
High	326	6.5
Don’t know	1,015	20.3
How often do you worry about getting breast cancer at some point?		
Rarely / never / don’t know	2,925	58.5
Sometimes	1,665	33.3
Almost always / often	411	8.2

^a^When sum of a category is less than 100%, the remaining data is “missing”

Two characteristics differed from the Danish general female population. The study population had higher level of education [22], and the screening participation rate was higher compared to the general female population [3]. The remaining demographic characteristics of the participants are comparable to the Danish female population aged 52 to 67 years.

### Danish women’s attitudes towards risk-stratified breast cancer screening

**Fig. 1:**
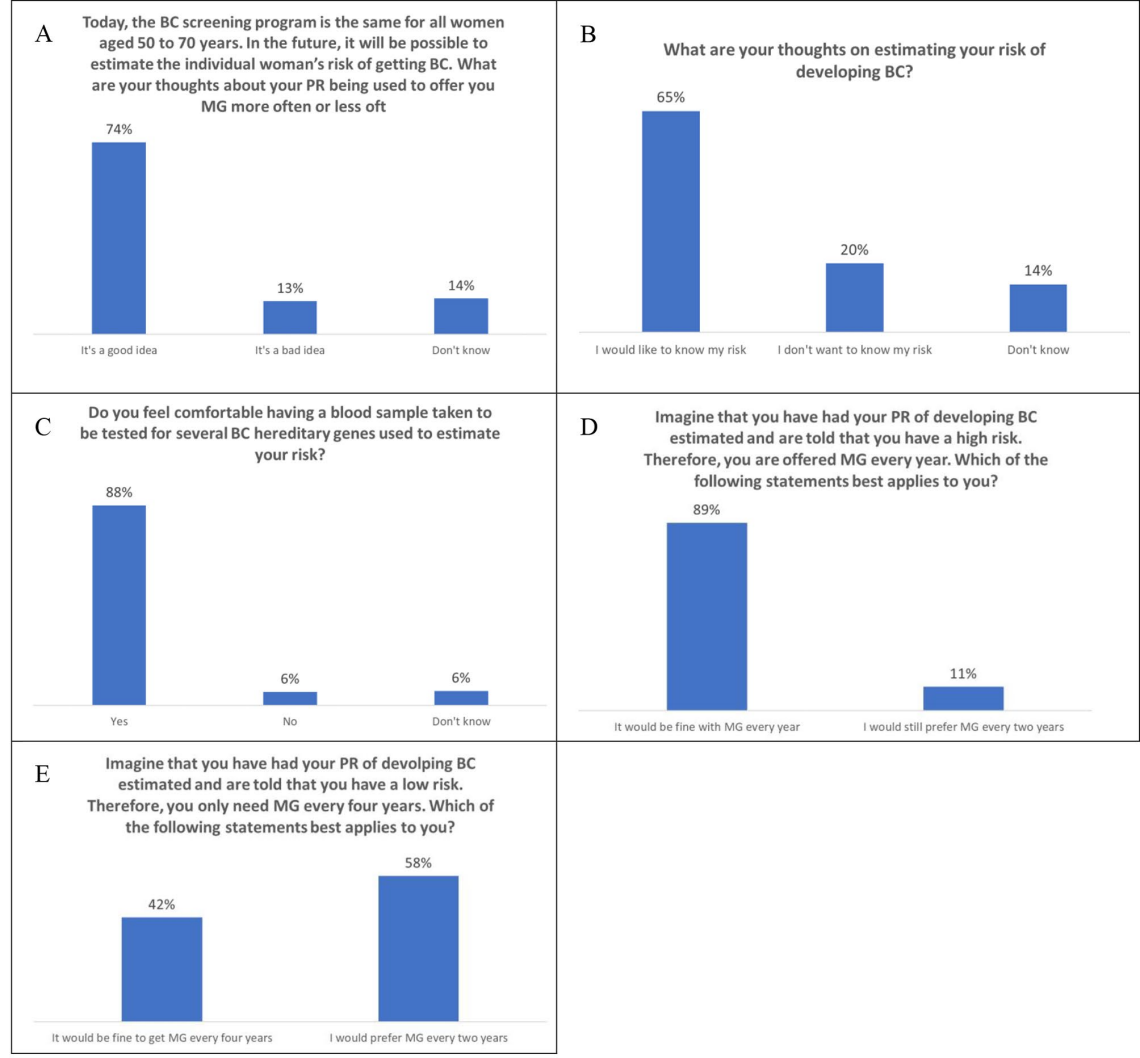
Attitudes towards risk-stratified breast cancer screening among 5,001 52- to 67-year-old Danish women. Abbreviations: PR = personal risk, BC = breast cancer, MG = mammography

Most women (74%) thought that it was a good idea to use estimates of personal risk to offer mammography either more or less frequently than today, while 13% did not and 14% responded “Don’t know” (Fig. 1A). Further, 65% of the respondents stated that they would like to know their personal risk of developing breast cancer (Fig. 1B). Their main reasons for wanting to know more about their personal risk were so that they could do something to reduce their risk (39%), feel safer (25%) and wanted only to be examined when necessary (22%) (data not shown). One out of five (20%) respondents stated that they do not want their personal risk estimated (Fig. 1B) mainly because they do not want to worry (64%) or feel sick (14%) (data not shown). Most women (88%) would feel comfortable having a blood sample taken, and only 6% stated that they would refuse (Fig. 1C).

Also, most women (89%) responded that they would feel fine about having a yearly screening for breast cancer if they were found to be at high risk (Fig. 1D), while less than half (42%) would be fine with screening every four years if they were found to be at low risk (Fig. 1E). When asked why they still preferred mammography every second year if found to be at low risk, respondents replied that they would be worried about the additional time passing between their screenings (51%), that it would feel like a degradation (32%) and that they would need more knowledge about the reason for changing the screening program (16%) (data not shown).

### Predictors of being negative or hesitant towards risk-stratified breast cancer screening

Table 2 shows that increasing age was associated with higher odds of being negative or hesitant about estimation of personal risk of breast cancer (OR = 1.03, 95% CI [1.02,1.04], *p* < 0.001) and about having a blood sample taken (OR = 1.05, 95% CI [1.03, 1.07], *p* < 0.001). Age was not associated with attitude to alter the screening interval if found to be at low risk (OR = 0.99, 95% CI [0.98-1.00], *p* = 0.07).

Women with medium or lower education had higher odds of being negative or hesitant about estimation of their personal risk (OR = 1.21, 95% CI [1.06–1.37], *p* < 0.003 and OR = 1.74, 95% CI [1.37, 2.21], *p* < 0.001) and having a blood sample taken (OR = 1.30, 95% CI [1.08–1.57], *p* < 0.005 and OR = 2.09, 95% CI [1.54, 2.82], *p* < 0.001) compared to women with high education. Women with medium or lower education also had higher odds of preferring to continue with mammography every second year, even if found to be at low risk of breast cancer, compared to women with high education (OR = 1.54, 95% CI [1.36–1.75], *p* < 0.001 and OR = 1.65, 95% CI [1.28, 2.13], *p* < 0.001) (Table 2).

Women who had not participated in breast cancer screening had higher odds of being negative or hesitant about estimation of personal risk (OR = 2.39, 95% CI [1.92–2.98], *p* < 0.001) and having a blood sample taken (OR = 3.55, 95% CI [2.76–4.56], *p* < 0.001) compared to screening participants. Non-participants had lower odds of preferring biannual screening if they were found to be at low risk compared to screening participants (OR = 0.17, 95% CI [0.13–0.22], *p* < 0.001) (Table 2).

Woman without personal or family history of breast cancer had higher odds of being negative or hesitant about estimation of personal risk (OR = 1.96, 95% CI [1.40–2.81], *p* < 0.001 and OR = 1.40, 95% CI [1.22–1.62], *p* < 0.001) and having a blood sample taken (OR = 2.59, 95% CI [1.48-5.00], *p* < 0.002 and OR = 1.26, 95% CI [1.03–1.56], *p* = 0.03) compared to women with a personal or family history of breast cancer. Not having a personal history of breast cancer was associated with lower odds of preferring screening biannual if they were found to be at low risk (OR = 0.66, 95% CI [0.47–0.91], *p* = 0.01). This also appeared to be the case for women with a family history of breast cancer; however, the association was only borderline significant (OR = 0.87, 95% CI [0.76-1.00], *p* = 0.05) (Table 2).

Women assessing their risk of breast cancer as low had higher odds of being negative or hesitant about estimation of personal risk (OR = 1.82, 95% CI [1.27–2.66], *p* < 0.002) compared to women who assessed their risk as high. The results indicated increased odds of being negative or hesitant about having a blood sample taken when assessing risk as low compared to high. However, this association was only borderline significant (OR = 1.88, 95% CI [1.04–3.73], *p* = 0.05). Assessing risk of breast cancer as “neither high nor low” or replying “Don’t know” increased the odds of being negative or hesitant about estimation of personal risk (OR = 2.93, 95% CI [2.07–4.22], *p* < 0.001), and having a blood sample taken (OR = 3.33, 95% CI [1.88–6.47], *p* < 0.001) compared to assessing their risk as high. Assessing breast cancer risk as low was associated with lower odds of preferring screening biannually if found to be at low risk compared to assessing risk as high (OR = 0.65, 95% CI [0.47–0.88], *p* < 0.007). Respondents’ attitudes towards altering their screening frequencies if found to be at low risk did not differ between women with high risk assessment and women replying “Neither high nor low” or “Don’t know” (OR = 1.15, 95% CI [0.84–1.54], *p* = 0.38) (Table 2).

Women with low breast cancer worry (responding “Rarely”, “Never” or “Don’t know”) had higher odds of being negative or hesitant about estimation of personal risk (OR = 1.36, 95% CI [1.05–1.76], *p* = 0.02) and lower odds of preferring biannual screening if they were found to be at low risk (OR = 0.24, 95% CI [0.18–0.32], *p* < 0.001) compared to women with high breast cancer worry (those responding “Almost always” or “Often”). Women who “sometimes” worried about getting breast cancer had lower odds of preferring screening every second year if at low risk compared to women with high breast cancer worry (OR = 0.57, 95% CI [0.42–0.76], *p* < 0.001). Breast cancer worry was not associated with attitude towards having a blood sample taken (OR = 1.09, 95% CI [0.76–1.60], *p* = 0.67 and OR = 1.05, 95% CI [0.72–1.56], *p* = 0.80) (Table 2).

### Sensitivity analyses

We performed sensitivity analyses, excluding those women who were hesitant about risk-stratified breast cancer screening from the models presented in Table 2. These analyses, presented in Additional file 2, demonstrate the same overall results as presented in Table 2. Excluding women who have or have had breast cancer also demonstrated the same overall results (data not shown).

**Table 2:** Multivariable logistic regression analyses for the association between three estimates of attitude towards risk- stratified breast cancer screening and characteristics of the study population (*n* = 5,001)

	What are your thoughts on estimating your risk of developing BC? I don’t want to know my risk/don’t know vs. I would like to know my risk^a^			Imagine that you have had your personal risk of developing BC estimated and are told that you have a low risk. Therefore, you only need MG every four years. Which of the following statements best applies to you? I would prefer MG every two years vs. It would be fine to get MG every four years^b^			Do you feel comfortable having a blood sample taken to be tested for several BC hereditary genes used to estimate your risk? No/don’t know vs. Yes^c^		
Characteristics	**OR** ^**d**^	**95% CI**	***P-value***	**OR** ^**d**^	**95% CI**	***P-value***	**OR** ^**d**^	**95% CI**	***P-value***
*Age (52–67)*	1.03	1.02–1.04	**< 0.001**	0.99	0.98-1.00	0.072	1.05	1.03–1.07	**< 0.001**
*Education level*									
High (< 10 years)	-1-	ref		-1-	ref		-1-	ref	
Medium (10–12 years)	1.21	1.06–1.37	**0.003**	1.54	1.36–1.75	**< 0.001**	1.30	1.08–1.57	**0.005**
Lower (> 12 years)	1.74	1.37–2.21	**< 0.001**	1.65	1.28–2.13	**< 0.001**	2.09	1.54–2.82	**< 0.001**
*Are you participating in the BC screening program, where you are invited every two years?*									
Yes	-1-	ref		-1-	ref		-1-	ref	
No	2.39	1.92–2.98	**< 0.001**	0.17	0.13–0.22	**< 0.001**	3.55	2.76–4.56	**< 0.001**
*Do you have or have you had BC?*									
Yes, I have/have had BC	-1-	ref		-1-	ref		-1-	ref	
No	1.96	1.40–2.81	**< 0.001**	0.66	0.47–0.91	**0.013**	2.59	1.48-5.00	**0.002**
*Does anyone in your immediate family have or have had breast cancer?*									
Yes, one or more members of my immediate family have/have had BC	-1-	ref		-1-	ref		-1-	ref	
No/Don’t know	1.40	1.22–1.62	**< 0.001**	0.87	0.76-1.00	0.052	1.26	1.03–1.56	**0.025**
*How do you assess your own risk of getting BC?*									
High	-1-	ref		-1-	ref		-1-	ref	
Low	1.82	1.27–2.66	**0.002**	0.65	0.47–0.88	**0.007**	1.88	1.04–3.73	0.051
Neither high nor low/Don’t know	2.93	2.07–4.22	**< 0.001**	1.15	0.84–1.54	0.377	3.33	1.88–6.47	**< 0.001**
*How often do you worry about getting BC at some point?*									
Almost always/often	-1-	ref		-1-	ref		-1-	ref	
Sometimes	1.07	0.80–1.35	0.785	0.57	0.42–0.76	**< 0.001**	1.05	0.72–1.56	0.804
Rarely/Never/Don’t know	1.36	1.05–1.76	**0.020**	0.24	0.18–0.32	**< 0.001**	1.09	0.76–1.60	0.668

^a^OR signifies the adjusted odds ratio of responding “I don’t want to know my risk” or “Don’t know” rather than “I would like to know my risk”, ^b^OR signifies the adjusted odds ratio of responding “I would prefer mammography every two years rather than “I would be fine to get mammography every four years”, ^c^OR signifies the adjusted odds ratio of responding “No” or “Don’t know” rather than “Yes”, ^d^OR is adjusted for all covariates in each of the three models (age, level of education, screening participation, personal and family history of breast cancer, breast cancer risk and breast cancer worry).

Abbreviations: BC = breast cancer, MG = mammography.

## Discussion

The study found that most women are positive about risk-stratified breast cancer screening, especially if they imagine being at high risk with the possibility to be offered more frequent screenings. Several determinants increase the odds of women being negative or hesitant: higher age, low level of education, low perceived breast cancer risk, no personal or family history of breast cancer, non-participation in breast cancer screening and low level of breast cancer worry. It is essential to address these determinants if a risk-stratified program is to be successfully implemented.

The respondents’ generally positive attitude towards estimation of personal risk (65%) is supported by qualitative interviews among Danish women [18]. These findings are also in line with studies from other countries, ranging from 74 to 94% of women showing interest in estimating their personal risk of breast cancer [12–16]. These findings are promising, since implementation of a risk-stratified screening program would require that women are willing to know their own risk. However, it is still essential to accommodate the 20% who state that they do not want to know their risk. In this study, the main reason for women being reluctant to have their personal risk estimated is that they do not want to worry, which is consistent with findings from the UK [14] and with our own findings that women with low levels of breast cancer worry were more reluctant towards risk estimation. This is further supported by a study demonstrating that breast cancer worry increases interest in genetic testing [23]. Consistent with other studies, we found that women of increasing age [12, 13, 16], low education [16], low perceived breast cancer risk [16] and women without a personal history of breast cancer [16] were more negative or hesitant about estimation of personal risk. It is encouraging that the vast majority of our respondents (88%) would feel comfortable having a blood sample taken, since willingness to provide a blood sample is crucial to estimate the risk profile that forms the basis for a risk-stratified breast cancer screening program. This finding is consistent with findings from Canada, Sweden, and Holland, where positive attitudes towards having a blood sample range from 67–98% [12, 15, 16]. Increasing age and lower education were associated with being more reluctant to have a blood sample taken, which is in line with findings from Canada and Sweden [15, 16]. Women reporting a breast cancer risk of “Neither high nor low” or responding “Don’t know” are less likely to want a blood sample taken. This group of woman are even more reluctant towards risk estimation than those reporting a low risk. This association between risk perception and health behavior is consistent with Protection Motivation Theory, by which high risk perception is a predictor of engaging in risk-reducing behavior [24]. When developing communication strategies, consideration should also be given to the influence of women’s risk perception.

Risk-stratified breast cancer screening holds great potential in terms of improving survival of the patients, preventing over-diagnosis and reducing costs. Simulation studies have shown that reducing the frequency of screening may benefit up to 25% of low-risk women with no screening at all after baseline [25]. However, these benefits can only be gained if women also accept less frequent screenings. We found that many women assessed as low risk would still be concerned about less frequent screenings, a finding consistent with other studies [14–17]. A Danish qualitative study even suggests that the potential to receive less frequent screenings is one of the main barriers to participation in the randomized trial PRSONAL [18]. Concerns about more time passing between screenings are supported by other studies, where fear of missed cancers is the major concern [14, 15]. We found that women with a lower level of education had increased odds of preferring screening biannually if found to be at low risk, which is consistent with findings from Sweden [15]. We also found that higher level of breast cancer worry and perceived risk was associated with higher odds of preferring screening biannually if the women were found to be at low risk. These results also emphasizes that higher risk perception predicts greater interest in risk-reducing behavior [24]. A Canadian study also found less favorable attitudes toward extending the screening interval among women with high risk perception [16]. In general, women who are unsure about participating in a risk-stratified screening program have a greater need for additional information [26]. Research among UK women suggests that acceptance of extending screening interval requires evidence-based information with a clearly explained rationale [27]. However, information about risk-stratified screening has proven to be difficult to understand for lay people [28]. In order to implement a successful risk-stratified program, effective communication strategies addressing barriers to extend screening interval should be further explored, as it will be fundamental to women’s acceptance of the program and thus to realize the projected benefits of implementing such a program.

Those women who are most negative or hesitant about estimation of their personal risk and having a blood sample taken have characteristics similar to those low-risk women who accept less frequent screening. These findings indicate that these women in general have a lower interest in screening rather than a negative attitude towards risk-stratified screening per se. As shown in Table 2, these women are more likely not to participate in screening, to be without a personal or family history of breast cancer, to have low perceived risk of breast cancer and low degree of breast cancer worry. These data suggest that screening has a low level of relevance for this group.

The results of this study indicate that implementation of risk-stratified breast cancer screening in Denmark will be positively received by most women, but only if they are sufficiently informed, although some women will continue to be reluctant to extend screening interval, have a blood sample taken or have their personal risk assessed, especially women with lower education and increasing age. This same group of women are already less likely to participate in the Danish breast cancer screening program [29]. In addition, we know that ethnic minorities have higher rates of non-participation [29]. Further research is needed in order to explore how implementation of a more complex concept as risk-stratified breast cancer screening could influence social inequalities in screening participation and to ensure that inequalities are not enhanced. Under real-world conditions of implementation, these considerations must be integrated into a decision tool with adaptable risk communication. Thus, the implementation of risk-stratified breast cancer screening can be affected, enhanced or constrained by local culture, infrastructure, and health care systems, in addition to the pathobiological insights and therapeutical possibilities.

A self-complete survey was an appropriate method for assessing women’s attitude towards risk-stratified breast cancer screening [30]. However, there are several limitations to this study. Although most women expressed positive views about risk-stratified screening, they have limited knowledge about the concept. Risk-stratified screening is complex, and in the survey we endeavored to explain it in as easy-to-understand manner as possible. However, research suggests that people with low health literacy, who are more likely to have a low education, may respond to questions even if they do not fully understand them [31]. Thus, respondents’ level of education may be associated with the degree of correct understanding of the survey questions. In this case, the results may have overestimated educational differences in attitudes towards risk-stratified breast cancer screening. A degree of caution is necessary when translating attitudes expressed in a hypothetical scenario into the real world behavior of an implemented program. Social desirability bias may have overestimated the degree of positive attitudes in the study. In addition, generalizability is somewhat compromised by a non-random sampling, and the self-selected respondents were more likely to have higher education and participate in screening than the general population. This may also have led to an overestimation of the women’s positive attitudes towards risk-stratified screening. However, we used quotas to ensure that the sample’s sociodemographic characteristics did not differ considerably from the wider female population of the same age group, thus making this study generalizable to the entire Danish female population. The large sample size of 5,001 women, a strength of this study, allowed us to explore associations between women’s attitudes and a range of other determinants.

## Conclusion

This study adds new insights into Danish women’s attitude towards risk-stratified breast cancer screening. We found that Danish women are positive towards the concept of risk-stratified breast cancer screening, especially if they imagine being at high risk and will then be offered more frequent screenings. Being negative or hesitant about risk-stratified screening is associated with higher age, lower education, no history of breast cancer, low level of breast cancer worry and low perceived risk of breast cancer. Implementation of risk-stratified breast cancer screening might benefit from starting with the youngest age group, while simultaneously developing clear communication strategies focusing on the subgroup of women who are more negative or hesitant towards changing screening frequencies, especially those with low breast cancer risk.

### Electronic supplementary material

Below is the link to the electronic supplementary material.

Supplementary Material 1

Supplementary Material 2

## Data Availability

Data supporting the results are available upon request to the corresponding author JB (Bigaard@cancer.dk).
