# Supplementary material for: Attitudes towards risk-stratified breast cancer screening: a population-based survey among 5,001 Danish women
Source: BMC Cancer. 2024 Mar 19;24:347. doi: 10.1186/s12885-024-12083-2 (PMC10949660; doi:10.1186/s12885-024-12083-2)
Supplement: Supplementary file 1 — Supplementary Material 1 [file 12885_2024_12083_MOESM1_ESM.docx]

**Questionnaire survey:**

Women's attitudes toward risk-based breast cancer screening and participating in a research project on breast cancer screening.

**Introduction**

*Information*

Presently in Denmark, all women between the ages of 50 and 70 are invited to have a breast cancer screening examination every two years. In the future, it will be possible to adapt the screening program to the individual woman's risk profile, so that she will be offered screening every year if she is at high risk, every two years if she is at medium risk, and every four years if she is at low risk.

From 2024, the Copenhagen Metropolitan Region will be offering such a program as part of a research project on one thousand women. In the project, half of the women will be placed in a group that continues with mammography every two years. The other half of the group will have their personal risk of developing breast cancer calculated and during the course of the project, these women will be offered screening at different frequencies based on their assessed risk. The two groups will be formed using a random lottery.

With this questionnaire, we examine women's attitudes to a screening program where the woman's risk of developing breast cancer determines whether she is offered mammography more or less frequently than is the case today. Our research project will also investigate women's attitudes about knowing their own risk of developing breast cancer.

Question 1:

Single Multi

Q1c Do you have or have you ever had breast cancer?

1. Yes, I have breast cancer

2. Yes, I have had breast cancer

3. No Exclusive

4. Don't want to answer Exclusive

Single Multi

Q1d Is there anyone in your immediate family who has or has had breast cancer?

1. Yes, one or more members of my immediate family have breast cancer

2. Yes, one or more members of my immediate family have had breast cancer

3. No Exclusive

4. Don't know Exclusive

5. Don't want to answer Exclusive

Single

Q1e Are you participating in the breast cancer screening program, where you are invited every two years?

1. Yes

2. No

Single

If Q1e=2

Q1e2 Why do you not participate in the breast cancer screening program?

1. I do not participate yet, but I expect to do so in the future

2. I do not wish to participate

Question 2:

Single

Q2 How often do you worry about getting breast cancer at some point?

1. Almost always

2. Often

3. Sometimes

4. Rarely

5. Never

6. Don't know

Question 3:

Single

Q3 How do you assess your own risk of getting breast cancer?

1. High

2. Low

3. Neither high nor low

4. Don't know

**1. Attitudes towards personal breast cancer screening**

Question 4:

Single

Q4 What do you think about the fact that knowledge about your age, lifestyle, breast cancer in the family, number of children, your weight, height and a blood test can be used to calculate whether you have a high or low your risk of breast cancer?

1. It's a good idea

2. It's a bad idea

3. Don't know

Question 5:

Single

Today, the breast cancer screening program is the same for all women aged 50 and 70 years. In the future, it will be possible to estimate the individual woman’s risk of getting breast cancer. What are your thoughts about your personal risk being used to offer you a mammography more often or less often than today?

• That’s a good idea

• That’s a bad idea

• Don’t know

*Information*

As previously mentioned, half of the women in this research project will be assigned to a group that continues with mammography every two years and does not have their personal risk calculated.

The other half will have their personal risk of developing breast cancer calculated, and during the project period they will be offered screening more or less frequently based on their personal risk. Random lots will be drawn to determine which group you will be in.

Question 6

Single

Q6 Which group would you prefer to join: the group that has its personal risk calculated, or the group that continues with breast cancer screening every two years without having their personal risk calculated?

1. The group that gets its personal risk calculated

2. The group that does not get its personal risk calculated

3. Don't know

Question 7:

Single

Q7 What are your thoughts on estimating your risk of developing breast cancer?

1. I would like to know my risk

2. I do not want to know my risk

3. Don't know

Single

If Q7=1

Q7a Which of the following statements best applies to you:

I would like to know my risk because:

1. Then I won’t feel so worried

2. Then I will feel more secure

3. Then I only have to be examined when necessary

4. Then I can do something to reduce my risk

5. Other: _ Open

6. Don't know

Single

If Q7=2

Q7b Which of the following statements best applies to you:

I do not want to know my risk because:

1. I don't want to feel worried

2. I don't want to feel that I am sick

3. I think that breast cancer strikes randomly anyway

4. We don't have breast cancer in the family, so it's not relevant for me

5. Other: Open

6. Don't know

**2. Attitudes to providing information and be examined in order to participate**

*Information*

In order to calculate your risk of breast cancer, in addition to your mammography, you will be required to answer a number of questions. You must also have a blood test that will be used to determine several genetic predispositions for breast cancer. This information will be stored securely.

Question 8:

Single

Q8 Would you be comfortable providing the following personal details that will be used to calculate your risk: questions about lifestyle, breast cancer in the family, number of children, when you got your first period, use of hormones, height and weight?

1. Yes

2. No

3. Don't know

Question 9:

Single

Q9 Do you feel comfortable having a blood sample taken to be tested for several breast cancer hereditary genes used to estimate your risk?

1. Yes

2. No

3. Don't know

**3. Willingness to participate in personal breast cancer screening**

Question 10

Single

Q10 Imagine that you have had your personal risk of developing breast cancer estimated and are told that you have a low risk. Therefore, you only need a mammography every four years. Which of the following statements best applies to you?

1. It would be fine to have get a mammography every four years

2. I would prefer having a mammography taken every two years

Single

If Q10=1

Q10a Which of the following statements best applies to you in connection with having a mammography every four years (based on your personal risk)?

1. It would be fine, because I would feel safe, since I expect that there is solid science behind it

2. It would be fine, because it is important to prioritize the resources in the healthcare system

3. It would be fine, because then I would be exposed to less X-ray radiation

4. It would be fine, because I would then have less discomfort and hassle with the mammographies

5. Other open

Single

If Q10=2

Q10b Which of the following statements best applies to you in connection with having a mammography every four years (based on your personal risk)?

1. I would be concerned that there was such a long time between the mammographies

2. It would feel like a worsening

3. I would need to have more information about why the program has been changed

4. Other open

Question 11

Single

Q11 Imagine that you have had your personal risk of developing breast cancer estimated and are told that you have a high risk. Therefore, you are offered a mammography every year. Which of the following statements best applies to you?

1. I would be fine with a mammography every year

2. I would still prefer having a mammography every two years

Single

If Q11=1

Q11a Which of the following statements best applies to you in connection with having a mammography every year (based on your personal risk)?

1. I would be concerned about being at high risk for breast cancer, but I would feel comfortable with a mammography every year

2. I would be happy that I am being closely monitored

3. It provides a better opportunity to detect cancer early

4. Other open

Single

If Q11=2

Q11b Which of the following statements best applies to you in connection with having a mammography each year (based on your personal risk)?

1. I would be concerned about being at high risk for breast cancer, but I would prefer having a mammography every two years, as I do now

2. It is not certain that I will ever develop breast cancer, so I would prefer having a mammography every two years, as I do now

3. I think that having several screenings makes women feel that they are sick

4. Other open

*Information*

In the Copenhagen Metropolitan Region, 1000 women will be invited to participate in the research project. They will be divided randomly into the two groups of 500 each, as previously described. One group will have their personal risk of developing breast cancer calculated. The women's risk of developing breast cancer will be divided divided into ‘low-’, ‘medium-’ or ‘high risk’, respectively. This risk assessment will then determine how frequently she will be offered screening. The other group will not have their breast cancer risk calculated.

Both groups are included in the project and are therefore asked to complete short questionnaires about quality of life during the course of the project.

It is important that as many people as possible participate during the entire duration of the project. However, participants will always have the option of withdrawing from the research project and continue their mammography as usual.

The total duration of the research project will be four years.

Question 12

Single

Q12 Imagine that you have joined the group where your personal risk has not been calculated, and that you are offered a mammography every two years and must complete a series of questionnaires. How likely is it that you will choose to remain in the project?

1. Very likely

2. Likely

3. Unlikely

4. Very unlikely

5. Don't know

Question 13

Single

Q13 Imagine that you have had your personal risk calculated and that you are told that you are in the group of women with low risk. Therefore, you only need a mammography every four years and must complete a series of questionnaires. How likely is it that you will choose to remain in the project?

1. Very likely

2. Likely

3. Unlikely

4. Very unlikely

5. Don't know

Question 14

Single

Q14 Imagine that you have received your assessment of your personal risk of developing breast cancer. You are told that you are in the group of women with high risk. You are therefore offered a mammography each year and must complete a number of questionnaires. How likely is it that you will choose to remain in the project?

1. Very likely

2. Likely

3. Unlikely

4. Very unlikely

5. Don't know

**4. Knowledge of risk factors**

Question 15

Single

Q15 If you were a participant in the project, would you like to know more about the possibility of influencing your risk?

1. Yes. I am interested in knowing more about the causes and about what I can do to avoid breast cancer

2. No. I have sufficient knowledge of the causes, and I know that I have to live healthy and move about

3. Don't know

**5. Final questions**

Question 16

Single

Q16 Would you like to participate in the research project described in this questionnaire? The research project lasts a total of four years.

1. Yes

2. No

3. Don't know

Question 17

Multi

If Q16=1

Q17 Why would you like to participate in the research project?

1. I would like to contribute to improving the screening program

2. I am curious about knowing know my risk

3. I would like to participate in a research project, and I think that it is important to contribute to new knowledge

4. Other: Open
