# Supplementary material for: Attitudes towards risk-stratified breast cancer screening: a population-based survey among 5,001 Danish women
Source: BMC Cancer. 2024 Mar 19;24:347. doi: 10.1186/s12885-024-12083-2 (PMC10949660; doi:10.1186/s12885-024-12083-2)
Supplement: Supplementary file 2 — Supplementary Material 2 [file 12885_2024_12083_MOESM2_ESM.docx]

**Additional file 2: Sensitivity analyses**

The sensitivity analyses presented in Table S1 demonstrate the results of excluding women hesitant toward risk stratified breast cancer screening from the regression analyses presented in Table 2 in main manuscript.

Two regression models presented in Table 2 are included in the sensitivity analyses. The third model did not entail a group of hesitant women.

**Table S1:** Multivariable logistic regression analyses for the association between three estimates of attitude towards risk stratified breast cancer screening and characteristics of the study population

|  | *What are your thoughts on estimating your risk of developing BC?*  I don’t want to know my risk  vs. I would like to know my risk^a^  (n=4,290) | | | *Do you feel comfortable having a blood sample taken to be tested for several BC hereditary genes used to estimate your risk?*  No vs. Yes^b^  (n=4,680) | | |
| --- | --- | --- | --- | --- | --- | --- |
| Characteristics | **OR^c^** | **95 % CI** | **P-value** | **OR^c^** | **95 % CI** | **P-value** |
| *Age (52-67)* | 1.03 | 1.01-1.05 | **<0.001** | 1.05 | 1.02-1.08 | **<0.001** |
| *Education level* |  |  |  |  |  |  |
| High (<10 years) | -1- | ref |  | -1- | ref |  |
| Medium (10-12 years) | 1.28 | 1.10-1.49 | **0.002** | 1.29 | 1.00-1.67 | **0.049** |
| Lower (>12 years) | 1.76 | 1.33-2.33 | **<0.001** | 1.88 | 1.22-2.83 | **0.003** |
| *Are you participating in the BC screening program, where you are invited every two years?* |  |  |  |  |  |  |
| Yes | -1- | ref |  | -1- | ref |  |
| No | 3.00 | 2.36-3.82 | **<0.001** | 5.23 | 3.85-7.04 | **<0.001** |
| *Do you have or have you had BC?* |  |  |  |  |  |  |
| Yes, I have/have had BC | -1- | ref |  | -1- | ref |  |
| No | 1.77 | 1.18-2.83 | **0.008** | 3.07 | 1.36-8.82 | **0.017** |
| *Does anyone in your immediate family have or have had breast cancer?* |  |  |  |  |  |  |
| Yes, one or more members of my immediate family have/have had BC | -1- | ref |  | -1- | ref |  |
| No/Don’t know | 1.26 | 1.06-1.49 | **0.008** | 1.27 | 0.95-1.70 | **0.110** |
| *How do you assess your own risk of getting BC?* |  |  |  |  |  |  |
| High | -1- | Ref |  | -1- | ref |  |
| Low | 1.99 | 1.27-3.25 | **0.004** | 1.81 | 0.84-4.53 | 0.161 |
| Neither high nor low/Don’t know | 2.94 | 1.91-4.73 | **<0.001** | 2.41 | 1.15-5.91 | **0.032** |
| *How often do you worry about getting BC at some point?* |  |  |  |  |  |  |
| Almost always/often | -1- | ref |  | -1- | ref |  |
| Sometimes | 1.35 | 0.96-1.92 | 0.087 | 1.26 | 0.73-2.32 | 0.432 |
| Rarely/Never/Don’t know | 1.73 | 1.25-2.45 | **0.001** | 1.26 | 0.74-2.31 | 0.417 |

^a^OR signifies the adjusted odds ratio of responding “I don’t want to know my risk” rather than “I would like to know my risk”, ^b^OR signifies the adjusted odds ratio of responding “No” rather than “Yes”, ^c^OR is adjusted for all covariates in each of the three models (age, level of education, screening participation, personal and family history of breast cancer, breast cancer risk and breast cancer worry).

Abbreviations: BC = breast cancer, MG = mammography
